# Supplementary material for: Non-Enzymatic Glucose Detection Based on NiS Nanoclusters@NiS Nanosphere in Human Serum and Urine
Source: Micromachines (Basel). 2021 Apr 5;12(4):403. doi: 10.3390/mi12040403 (PMC8067435; doi:10.3390/mi12040403)
Supplement: Supplementary file 1 [file micromachines-12-00403-s001.pdf]

SUPPORTING INFORMATION

Non-Enzymatic Glucose Detection Based on NiS Nanoclusters @ NiS Nanosphere in Human Serum and Urine

Mani Arivazhagan, Yesupatham Manova Santhosh, and Govindhan Maduraiveeran*

Materials Electrochemistry Laboratory, Department of Chemistry, SRM Institute of Science and Technology, Kattankulathur, Tamil Nadu 603 203, INDIA

Corresponding Author E-mail: maduraig@srmist.edu.in

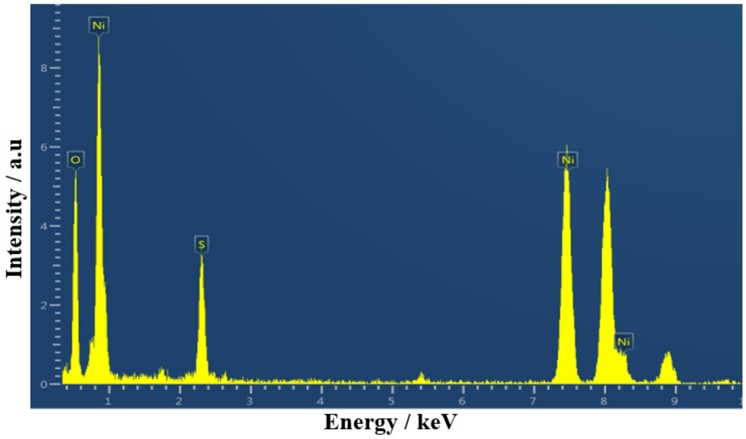

Fig. S1. EDX spectrum of the obtained NC-NiS@NS-NiS|NF.

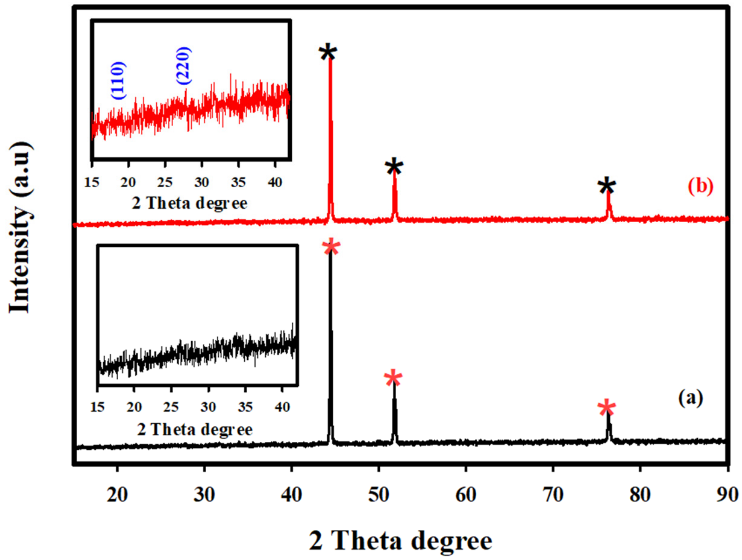

Fig. S2. XRD patterns of bare NF and NC-NiS@NS-NiS|NF electrodes.

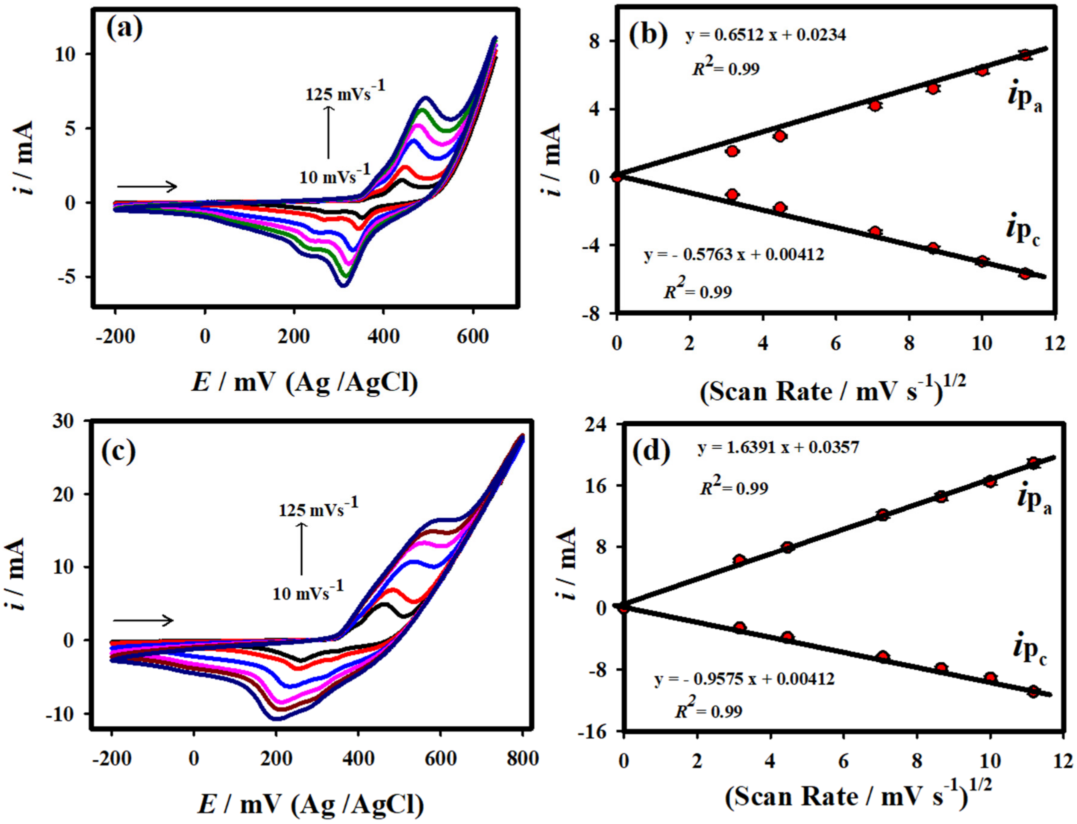

Fig. S3. CV curves of the NF (a), NC-NiS@NS-NiS|NF (c) recorded in 1.0 M KOH at different scan rates. The corresponding linear plot of $i\mathrm{p_a}$ and $i\mathrm{p_c}$ for bare NF (b) and NC-NiS@NS-NiS|NF electrodes (d).

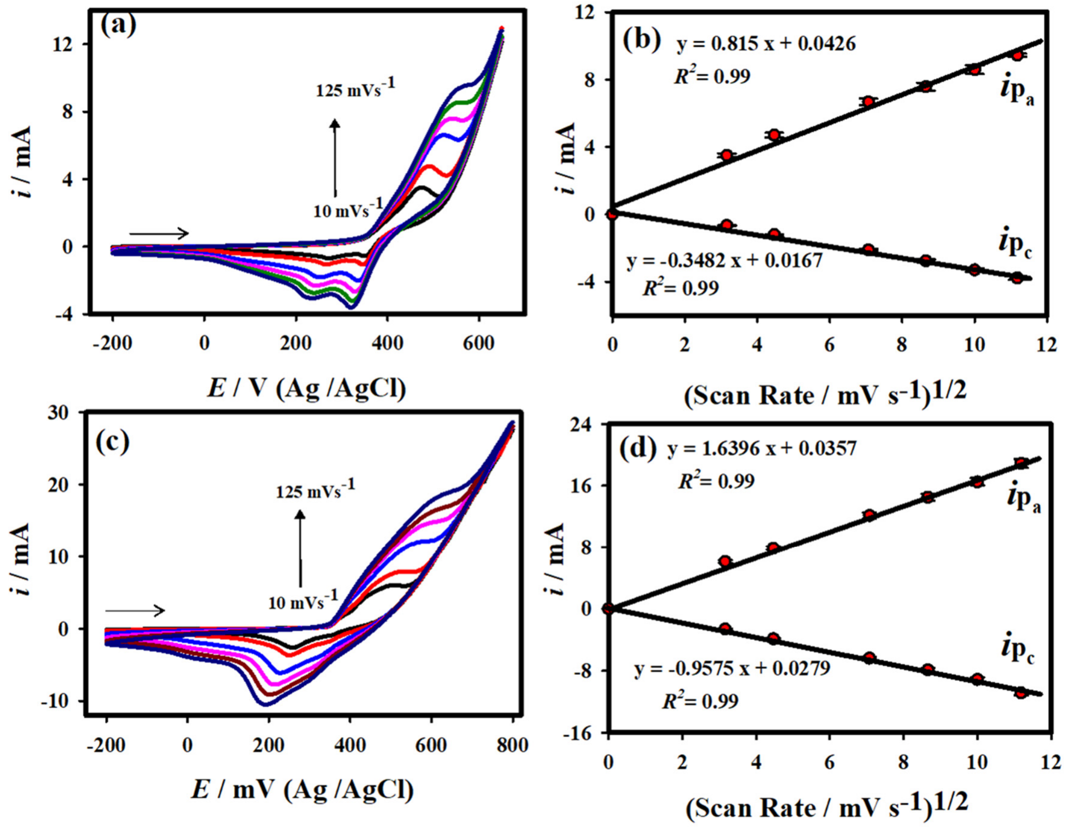

Fig. S4. CV curves of the NF electrode (a) and NC-NiS@NS-NiS|NF electrode (c) recorded in 10.0 mM glucose + 1.0 M KOH at different scan rates. The corresponding linear plot of ip_a and Ip_c for bare NF (b) and NC-NiS@NS-NiS|NF electrodes (d).

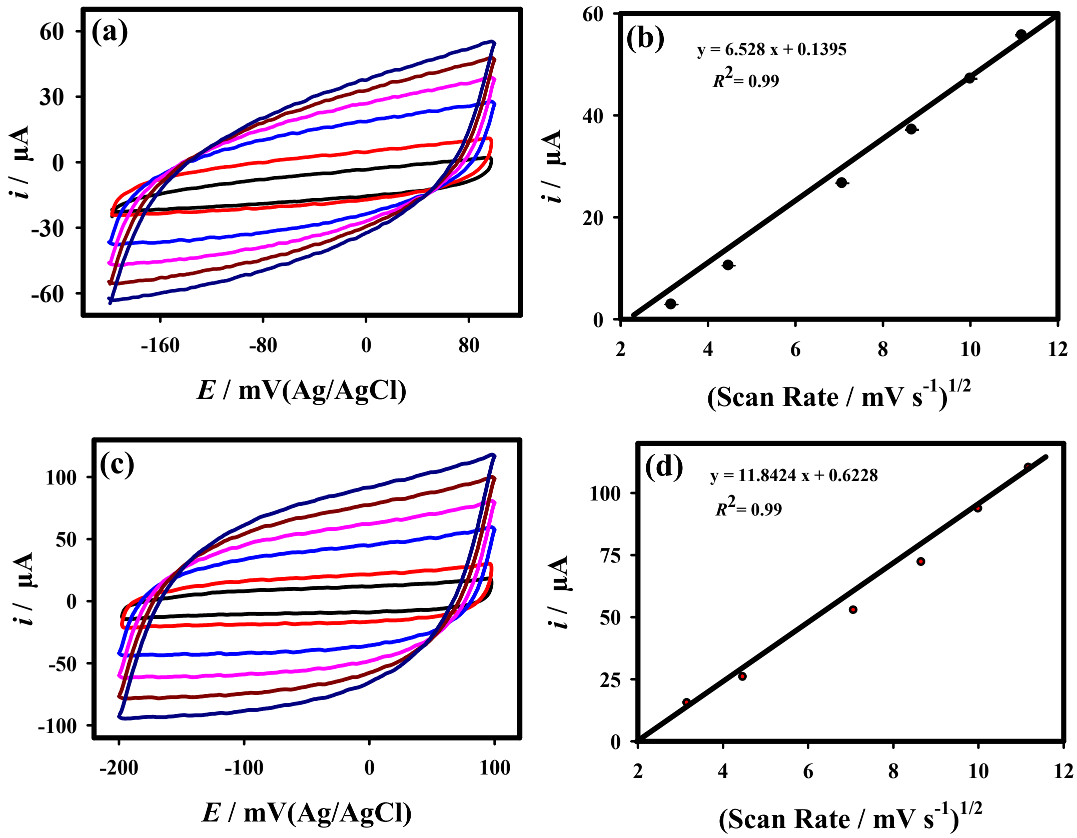

Fig. S5. (a) CV curves of the bare NF (a), and NC-NiS@NS-NiS|NF (c) electrodes recorded at various scan rates, starting from 10 mV s^{-1} to 125 mV s^{-1} in 1.0 M KOH. (b) The plot of anodic currents *vs* the square root of the scan rates for bare NF (b), and NC-NiS@NS-NiS|NF (d) electrodes.

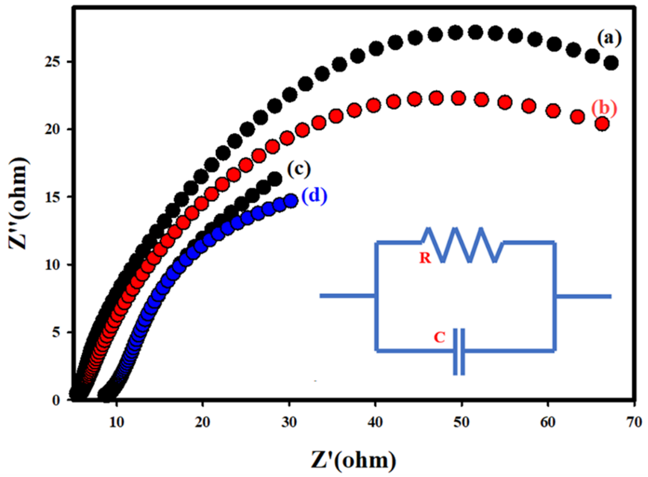

Fig. S6. EIS spectra of the bare NF and NC-NiS@NS-NiS|NF electrodes without glucose (black curve) and with 10 mM of glucose (red curve for bare NF & blue curve for NC-NiS@NS-NiS|NF electrodes).
